# New Insights into Parthanatos as Programmed Cell Death During Murine Cytomegalovirus or Herpes Simplex Virus Type 1 Productive Replication in Diverse Cell Types

**DOI:** 10.3390/cells15111009

**Published:** 2026-05-30

**Authors:** Jay J. Oh, Xinge Xie, Richard D. Dix

**Affiliations:** 1 Viral Immunology Center, Department of Biology, Georgia State University, Atlanta, GA 30303, USA; jay.oh@csmc.edu (J.J.O.); xxie8@gsu.edu (X.X.); 2 Department of Ophthalmology, Emory University School of Medicine, Atlanta, GA 30322, USA

**Keywords:** programmed cell death, parthanatos, murine cytomegalovirus, herpes simplex virus type 1

## Abstract

Programmed cell death (PCD) pathways of innate immunity serve to protect host cells from invading viruses. Parthanatos is a novel form of PCD triggered by excessive host cell DNA damage that leads to overactivation of poly(ADP-ribose) polymerase-1 (PARP-1) which in turn stimulates poly(ADP-ribose) (PAR) polymer formation. PAR translocates to the cytoplasm, where it induces release of apoptosis-inducing factor (AIF) from mitochondria, that then travels back to the nucleus, where it mediates large-scale DNA fragmentation and cell death. Little information is available regarding parthanatos as a cell death mechanism to dampen herpesvirus replication at the host cell level. A series of studies were therefore performed to clarify a possible role for parthanatos during productive replication of murine cytomegalovirus (MCMV) and herpes simplex virus type 1 (HSV-1) in diverse cell types. These included mouse embryo fibroblasts, mouse lung fibroblasts, mouse microglial (BV-2) cells, and human retinal pigment epithelial (ARPE-19) cells. We report that PAR protein production is surprisingly cell type specific. Moreover, MCMV or HSV-1 infection may suppress parthanatos as observed for other PCD pathways, such as apoptosis, necroptosis, and pyroptosis, in a dose-dependent and cell type-specific manner. We conclude that the operation of parthanatos at the host cell level during herpesvirus replication is more complex than originally thought but offers new targets for possible therapeutic interventions.

## 1. Introduction

A common feature of all disease states is cellular dysfunction that often progresses to cell death. Many mechanisms have been identified whereby a cell may undergo death. Among these are programmed cell death (PCD) signaling pathways of innate immunity that have gained considerable attention in recent years. Since the recognition of apoptosis as a form of PCD in 1972 [1], at least 15 additional forms of regulated cell death have been identified [2]. These include inflammatory forms of cell death such as pyroptosis [3] and necroptosis [4]. A summary of apoptosis, pyroptosis, and necroptosis signaling pathways and their representative signaling components is shown in Table 1.

**Figure 1:**
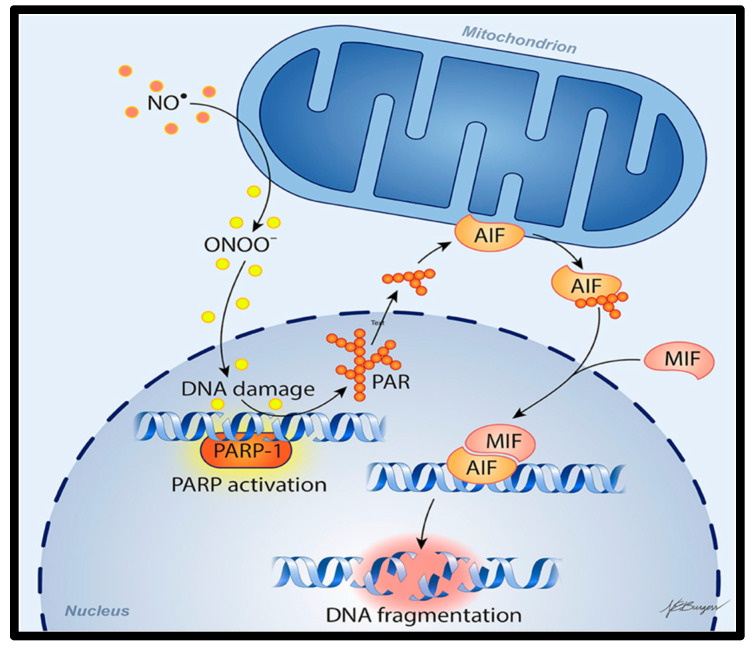
Diagram of parthanatos signaling. Nitric oxide (NO), pro-oxidant (ONOO), and other reactive oxygen species such as H_2_O_2_ cause extensive DNA damage that stimulates excessive activation of poly(ADP-ribose) polymerase (PARP-1) and abundant formation of poly(ADP-ribose) (PAR) polymers. PAR polymers translocate from the nucleus to the cytoplasm and cause the release of apoptosis-inducing factor (AIF) from the outer membrane of mitochondria. Upon release from the mitochondria, AIF may bind to macrophage migration inhibitory factor (MIF) and together enter the nucleus to produce large-scale DNA fragmentation and cell death. Adapted from Koehler, Dawson, and Dawson [14].

Parthanatos is a PCD signaling pathway first recognized in 2006 [5]. Its name is derived from *Thanatos*, the personification of death in Greek mythology, and PAR, a molecule key to its operation [6]. This novel form of cell death is mediated by the activation of poly(ADP-ribose) polymerase-1 (PARP-1), an enzyme routinely involved in the detection and repair of mild DNA damage. However, oxidative stress induced by reactive oxygen species (ROS) or other stimuli can lead to extensive DNA damage. This triggers excessive activation of PARP-1 that catalyzes the synthesis of poly(ADP-ribose) (PAR), a long-chain polymer with ribose sidechains that varies greatly in length. The more severe the DNA damage, the greater the accumulation of PAR in the nucleus. PAR then translocates from the nucleus to mitochondria of the cytoplasm, where it binds to apoptosis-inducing factor (AIF). Upon its release from mitochondria into the cytoplasm, AIF translocates from the cytoplasm to the nucleus. Here, cell death is induced through chromatin condensation and large-scale (50 Kb) DNA fragmentation [7,8,9,10,11,12,13] (Figure 1).

A detailed comparison of parthanatos with apoptosis, necroptosis, and pyroptosis is also shown in Table 1. Due to the apparent similarities in the mechanisms of operation for apoptosis and parthanatos, it is noteworthy that like apoptosis, cells undergoing parthanatos also exhibit chromatic condensation and DNA fragmentation although to a far greater extent than apoptosis. Unlike apoptosis, however, cells undergoing parthanatos exhibit neither apoptotic bodies, mitochondrial swelling, nor membrane blebbing [11,15]. Moreover, unlike apoptosis that is caspase dependent, parthanatos is caspase independent. Thus, parthanatos can be viewed as a unique PARP-1-dependent, caspase-independent cell death cascade that is AIF-mediated.

PCD serves many roles. Some are essential in maintaining normal physiologic homeostasis as occurs in the developing organism through the elimination of superfluous cells during embryonic morphogenesis, especially during neural development [16]. Other roles include the elimination of autoreactive immune cells and cancerous cells in the adult [17]. Of particular significance, however, is the role of PCD as a network of signaling pathways of innate immunity whose mission is to protect the host from invading pathogens, including resistance to virus infection. For example, several PCD pathways such as apoptosis have been recognized to prevent virus spread, thereby dampening tissue pathology and clinical disease onset through the elimination of virus-infected cells before active productive replication and release of progeny infectious viruses can occur. Other PCD pathways such as pyroptosis and necroptosis eliminate virus-infected cells through stimulation of a strong inflammatory response to virus infection.

Much of our knowledge regarding the precise roles of PCD pathways in antivirus host defense has come from studies of herpesviruses. Of these, human cytomegalovirus (HCMV) [18], murine cytomegalovirus (MCMV) [18], and herpes simplex virus type 1 (HSV-1) [19] have received considerable attention vis-à-vis apoptosis, pyroptosis, and/or necroptosis signaling pathways. In comparison, little information is available regarding a possible role for parthanatos as a cell death mechanism to dampen either HCMV, MCMV, or HSV-1 replication at the host cell level. Herein, we fill this knowledge gap by the performance of studies to further clarify a role for parthanatos during MCMV replication or HSV1 replication in several diverse cell types. Included among these cell types were mouse embryo fibroblasts (MEF cells) and mouse lung fibroblasts (MLg cells), which are historically known to be susceptible to MCMV infection and representative of cell types of clinical importance in humans during HCMV infection [20]. We also included mouse microglial cells (BV-2 cells) [21] and human retinal pigment epithelial cells (ARPE-19 cells) [22], two cell types found within retinal tissues. We were particularly interested in retinal cell types because we have previously shown that parthanatos may operate within the ocular compartment of retrovirus-immunosuppressed (MAIDS) mice during the onset and development of experimental MCMV retinal necrosis [23], a mouse model for AIDS-related HCMV retinal necrosis [24,25,26].

## 2. Materials and Methods

### 2.1. Cell Lines

Mouse embryo fibroblasts (MEF) (CRL-2991), mouse lung (MLg) fibroblasts (CCL-206), ARPE-19 cells (CRL-2302), human fibroblasts (MRC-5) (CCL-171), and African Green monkey kidney cells (Vero) (CCL-81) were obtained from the American Type Culture Collection (Manassas, VA, USA). Mouse microglial cells (BV2) (ABC-TC212S) were obtained from AcceGen Biotech (Fairfield, NJ, USA). All cell lines were grown and maintained in Dulbecco’s modified eagle medium (DMEM) supplemented with 10% fetal bovine serum (FBS), 4 mM L-glutamine, 1% penicillin/streptomycin, 0.1 mg/mL gentamicin, and 1.5 g/L sodium bicarbonate. Stimulation of oxidation stress in some cell types was accomplished by treating the uninfected cell monolayer with H_2_O_2_ (500 uM) contained in DMEM for 15 min prior to harvesting [27].

### 2.2. Viruses

All MCMV experiments were performed using the Smith strain of MCMV. Virus stocks were prepared in salivary glands of adult female BALB/c mice (Jackson Laboratory, Bar Harbor, ME, USA) following intraperitoneal injection of 10^2^ to 10^3^ PFU of virus. Salivary glands were harvested two weeks later and processed as described previously [23]. Quantification of virus stocks was accomplished by standard plaque assay using monolayers of MEF cells. Mice were housed in the AAALAC-accredited Georgia State University (GSU) vivarium, with unrestricted access to standard diet and water and maintained on alternative 12 h light-dark cycles. Use of BALB/c mice for preparation of virus stocks for these investigations was approved by the GSU Institutional Animal Care and Use Committee (IACUC) (Protocol A25033).

UV-inactivated MCMV (UVi-MCMV) was prepared as described previously [28]. Briefly, a 1 mL aliquot of infectious MCMV stock to be used in the same experiment was placed in an uncovered dish and exposed to DNA-damaging UV light for three hours at ~5 cm from the UV lamp. Complete inactivation of UVi-MCMV infectivity was confirmed on MEF monolayers by failing to show even one infectious virus plaque at 5 days after inoculation.

HSV-1 experiments were performed using the F strain, KOS63 strain, H299 strain, and H129 strain of HSV-1 [29,30]. Virus stocks were prepared in monolayers of MRC-5 human fibroblasts and quantified using Vero cells in a standard plaque assay as described previously [31].

### 2.3. Subcellular Fractionation

For detection of possible translocation of AIF from mitochondria of the cytoplasm to the nucleus, monolayers of MEF cells and MLg cells were infected with MCMV (moi = 10 PFU/cell) or mock infected. At 1, 2, and 3 days postinfection, all monolayers were harvested and 1 × 10^6^ cells of each experimental or control group were analyzed using a standard subcellular fractionation protocol to yield nuclear, mitochondrial, and cytosol fractions [32]. This was accomplished using a cell fractionation kit (ab109719) (Abcam Inc., Waltham, MA, USA) in accordance with the manufacturer’s instructions. Detection of parthanatos-associated proteins (PAR protein, PARP-1 protein, and AIF protein) in nuclear, mitochondrial, and cytosol fractions was accomplished by standard Western blot analysis.

### 2.4. Western Blot Assay

MCMV-infected and mock-infected cells upon harvest were lysed in Pierce IP lysis buffer (Thermo Fisher Scientific, Waltham, MA, USA) containing a protease inhibitor cocktail (Sigma, St. Louise, MO, USA). The protein concentration in each cell lysate was then determined by Bradford assay (Bio-Rad, Hercules, CA, USA). After normalization of protein concentrations, proteins were separated by electrophoresis in 8 or 10% sodium dodecyl sulfate-containing polyacrylamide gels and transferred to nitrocellulose membranes (GE Healthcare Life Sciences, Marlborough, MA, USA). Membranes were blocked for 1 h in Tris-Buffered Saline containing 0.1% Tween (TBST) and 5% skim milk powder (Thermo Fisher Scientific, Waltham, MA, USA), and all antibodies were diluted in TBST with 5% milk. Primary antibodies used for Western blot analysis in this investigation included rabbit anti-mouse PARP-1 antibody (1:1000) (ab191217) (Abcam Inc.), rabbit anti-mouse PAR antibody (1:500) (ALX-804-220-R100) (Enzo Life Sciences, Inc., Farmington, NY, USA), rabbit anti-mouse AIF antibody (1:500) (ab32516) (Abcam Inc.), rabbit anti-mouse β actin antibody (1:1000) (ab8227) (Abcam Inc.), and rabbit anti-mouse glyceraldehyde-3-phosphate dehydrogenase (GAPDH) (1:1000) (sc-137179) (Santa Cruz Biotechnology, Santa Cruz, CA, USA). After 1 h incubation with goat anti-rabbit polyclonal IgG antibody (1:5000) (31480) (Invitrogen, ThermoFisher Scientific, Waltham, MA, USA) (Heavy plus light chains [H + L]) or goat anti-mouse IgG antibody (1:5000) (115-035-003) (Jackson ImmunoResearch, West Grove, PA, USA) (Heavy plus Light chains [H + L]), the protein bands within the nitrocellulose membrane were visualized by chemiluminescence using an enhanced chemiluminescent (ECL) detection system (Bio-Rad, Hercules, CA, USA) and exposed to autoradiograph film (Thermo Scientific, Swedesboro, NJ, USA).

## 3. Results

### 3.1. PAR Protein Is Stimulated Within MCMV-Infected MEF Cells and MCMV-Infected MLg Cells in a Cell Type-Dependent Manner

**Figure 2:**
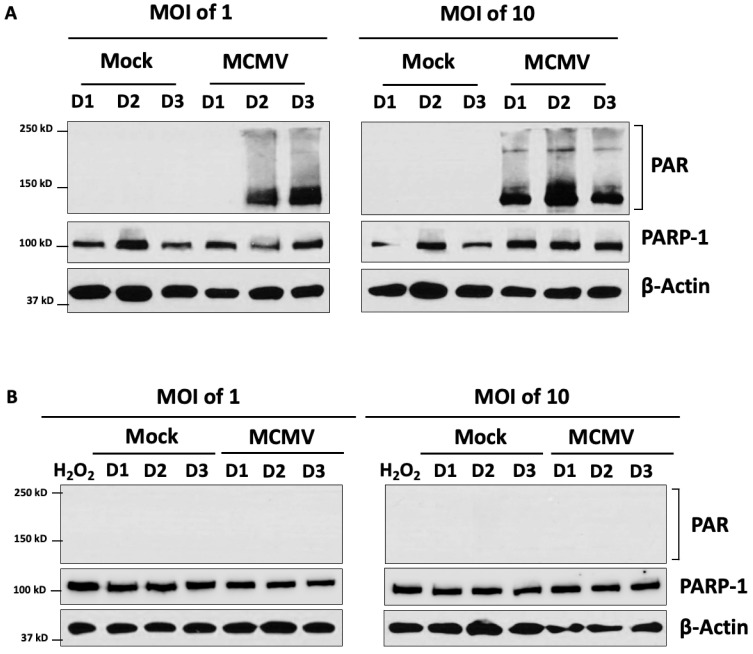
Monolayers of (**A**) mouse embryo fibroblasts (MEF cells) and (**B**) mouse lung fibroblasts (MLg cells) were infected with MCMV at moi = 1 PFU/cell or moi = 10 PFU/cell, or were mock-infected with cell culture maintenance medium (control). At 1, 2, and 3 days postinfection, monolayers were collected and assessed by Western blot analysis for detection of PAR protein, PARP-1 protein, and β-Actin protein (internal control). Uninfected MLg cells were treated with H_2_O_2_ (500 uM) contained in DMEM for 15 min prior to harvesting to induce oxidative stress [27] and PAR protein as a positive control.

Initial experiments were performed to assess and compare MEF cells and MLg cells for their ability to stimulate the production of PARP-1 proteins and PAR proteins following MCMV infection. Monolayers of MEF cells and MLg cells were inoculated with MCMV at input multiplicities of infection (moi) of either 1 PFU per cell or 10 PFU per cell; parallel monolayers for each cell type were mock-infected and served as controls. At 1, 2, and 3 days postinfection, virus-infected and mock-infected cell monolayers for each cell type were harvested and subjected to Western blot analysis for detection of PARP-1 protein and PAR protein. Results are shown in Figure 2.

As expected, PARP-1 protein was detected in both MCMV-infected and mock-infected MEF cells and MLg cells at all times examined, as this DNA repair enzyme is ever present in the nucleus of eukaryotic cells as a DNA damage sensor. This was not the case for PAR protein, as this was detected as a polymer with a wide range of molecular weights in MCMV-infected MEF but not in parallel mock-infected cells. In sharp contrast, PAR protein was not detected in MCMV-infected MLg cells at any of the postinfection times examined. Surprisingly, PAR protein was also not detected in H_2_O_2_-treated uninfected MLg cells that should serve as a positive control for the induction of oxidative stress and, therefore, parthanatos [27], as shown by us in a later experiment for H_2_O_2_-treated uninfected ARPE-19 cells (Figure 6).

Our results also suggest that PAR protein was also expressed in MCMV-infected MEF cells in a dose-dependent manner. Although not detected at 1 day postinfection in MCMV-infected MEF cells infected at an moi of 1 PFU per cell, PAR protein was readily detected at 1 day postinfection in MCMV-infected MEF cell inoculated with ten times more infectious virus.

### 3.2. PAR Protein Is Not Stimulated in MEF Cells Following Inoculation with UV-Inactivated MCMV

A possible role for virus-encoded protein during the induction of parthanatos via ROS by cytomegaloviruses remains unclear. This possibility was first suggested by Kim et al. [33] during HCMV infection of immunocytes, who traced PARP-1-induced parthanatos to at least four virus-encoded proteins, ranging from immediate-early proteins and a structural phosphoprotein. To shed further light on a possible role for MCMV-encoded proteins during the induction of parthanatos, we performed experiments using MCMV exposed to UV light (UVi-MCMV). This experimental approach renders virus as deficient in gene expression due to extensive damage to the virus genome, ultimately blocking productive replication [34]. Nonetheless, UV-inactivated virus undergoes attachment, adsorption, and release of tegument proteins into the host cell that would include several virus-encoded phosphoproteins.

**Figure 3:**
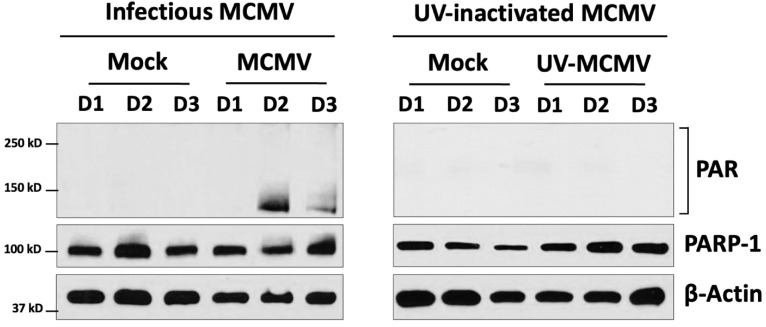
Monolayers of mouse embryo fibroblasts (MEF cells) were mock-infected with cell culture maintenance medium (control), infected with MCMV at moi = 1 PFU/cell, or inoculated with an equal volume of UV-inactivated MCMV (UVi-MCMV). At 1, 2, and 3 days postinfection, monolayers were collected and assessed by Western blot analysis for detection of PAR protein, PARP-1 protein, and β-Actin protein (internal control).

Because MEF cells were found to induce the PAR protein during MCMV replication (Figure 2A), monolayers of MEF cells were either mock-infected, inoculated with infectious MCMV (moi = 1), or inoculated with an equal amount of UVi-MCMV. At 1, 2, and 3 days postinfection, all monolayers were harvested and compared for detection of PARP-1 protein and PAR protein by Western blot analysis. As expected, PARP-1 protein was detectable at all times examined in mock-infected MEF cells, as well as in MEF cells inoculated with either infectious MCMV or UVi-MCMV (Figure 3). However, unlike MEF cells inoculated with infectious MCMV, PAR protein was not detected in MEF cells inoculated with UV-inactivated virus. These findings suggest that the release of MCMV-encoded tegument proteins into MEF cells during adsorption plays no role in the stimulation of PAR protein, or at least in MCMV-infected MEF cells.

### 3.3. PAR Protein Is Stimulated Within MCMV-Infected BV-2 Microglial Cells but in a Virus Dose-Dependent Manner

**Figure 4:**
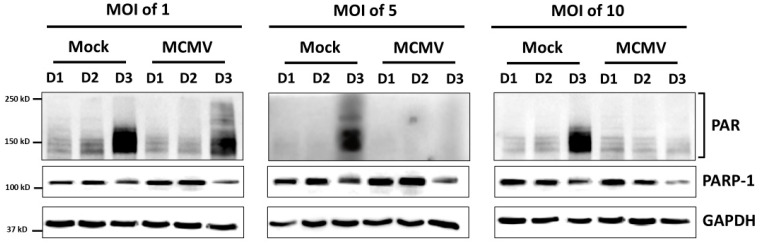
Monolayers of BV-2 mouse microglial cells were infected with MCMV at moi = 1 PFU/cell, moi = 5 PFU/cell, or moi = 10 PFU/cell, or were mock-infected with cell culture maintenance medium (control). At 1, 2, and 3 days postinfection, monolayers were collected and assessed by Western blot analysis for detection of PAR protein, PARP-1 protein, and GAPDH protein (internal control).

We next compared the pattern of PAR protein synthesis for MCMV-infected MEF cells with MCMV-infected BV-2 microglial cells, because microglial cells are constituents of retinal tissue that serve as resident innate immune cells of the retina [21]. Monolayers of BV-2 cells were either mock-infected or infected with MCMV at input virus doses of either 1, 5, or 10 PFU per cell. At 1, 2, and 3 days postinfection, virus-infected and mock-infected BV-2 cells were harvested and subjected to Western blot analysis for detection of PAR protein and PARP-1 protein. For these experiments, we also included monolayers of MEF cells infected with MCMV at moi of 1 PFU per cell and harvested 3 days postinfection as a positive control, because PAR protein was stimulated within MCMV-infected MEF cells at this time point and at this input virus dose, as previously found (Figure 2 and Figure 3). As shown in Figure 4, a pattern for PAR protein stimulation was observed for MCMV-infected BV-2 cells that was unlike that observed for MCMV-infected MEF cells. Whereas PAR protein production was detected in MCMV-infected BV-2 cells at moi of 1 PFU per cell, increased amounts of input infectious virus at doses of 5 and 10 PFU per cell failed to induce detectable amounts of PAR protein in repeated experiments. These findings suggest that MCMV might suppress the operation of parthanatos in BV-2 microglial cells but at a threshold amount of infectious virus greater that 1 PFU/cell. Nonetheless, like MCMV-infected MEF cells, PARP-1 was consistently detected in both mock-infected and MCMV-infected BV-2 cells at all times examined. However, unlike mock-infected MEF cells that showed no detectable PAR protein production, mock-infected BV-2 cells consistently showed background PAR protein production at 3 days after mock infection even though PAR protein was not detected at 3 days after infection of 5 or 10 PFU of MCMV. The reason for this unexpected observation remains unclear but may suggest that this microglial cell type is particularly sensitive to parthanatos stimulation under culture conditions that are abrogated by active virus replication in MCMV-infected cultures.

### 3.4. Nuclear Translocation of AIF Is Stimulated in Both MEF Cells and MLg Cells During MCMV Replication

**Figure 5:**
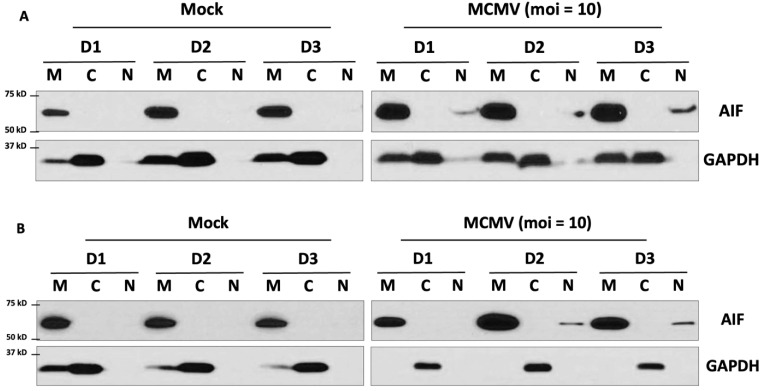
Monolayers of (**A**) mouse embryo fibroblasts (MEF cells) and (**B**) mouse lung fibroblasts (MLg cells) were infected with MCMV at moi = 10 PFU/cell or mock-infected with cell culture maintenance medium (control). At 1, 2, and 3 days postinfection, all monolayers were collected, subjected to subcellular fractionation [32], and the resulting mitochondrial (M), cytosolic (C), and nuclear (N) fractions were assessed by Western blot analysis for detection of AIF protein and GAPDH protein (internal control).

As shown in Figure 1, the parthanatos pathway is complex and involves several parthanatos-associated proteins that translocate to and from several compartments of the cell that include nucleus, mitochondria, and cytoplasm. Our initial investigations focused on early events in the parthanatos pathway, specifically a comparison of PARP-1 protein and PAR protein, in several cell lines. Downstream of these early events, however, is the need for AIF protein to be released from mitochondria and subsequently translocate to the nucleus to help initiate cell death by chromatin condensation and large-scale DNA fragmentation. To investigate possible parthanatos-associated AIF protein translocation from mitochondria to nucleus during MCMV replication, we performed cell fractionation studies for detection of AIF protein in mitochondria, nucleus, and cytosol during active replication of MCMV in MEF and MLg cell types. Monolayers of MEF cells and MLg cells were mock-infected or infected with MCMV (moi = 10), harvested at 1, 2, and 3 days postinfection, subjected to subcellular fractionation [32], and the resulting subcellular fractions enriched for mitochondria, cytosol, and nuclear contents were analyzed by Western blot for detection of AIF protein. Results are shown in Figure 5. AIF protein was detected in mitochondrial fractions of both mock-infected and virus-infected MEF and MLg cell types at all times examined as expected. Consistent with the operation of the parthanatos pathway was the detection of AIF protein in nuclear fractions of MCMV-infected MEF (but not in nuclear fractions of mock-infected cells) at 2 and 3 days postinfection. This observation was also in agreement with detection of PAR protein in MEF cells following MCMV infection (Figure 2). However, AIF protein was surprisingly also detected in nuclear fractions of MCMV-infected MLg cells at 2 and 3 days postinfection despite PAR protein not being detected during MCMV replication in this cell type. These findings suggest that MCMV replication induces the possible translocation of AIF protein from mitochondria to nucleus in both cell types (MEF and MLg) even though one cell type (MLg) exhibits no detectable stimulation of the PAR protein.

### 3.5. PAR Protein Is Not Stimulated in ARPE-19 Cells Infected with Any of Four Different Strains of HSV1

Having shown different outcomes for parthanatos-associated PAR protein production following MCMV infection of different mouse cell types, we performed experiments to compare these findings with those obtained for another herpesvirus, HSV-1, following infection of human ARPE-19 cells, a cell line of retina tissue origin [22]. We also included in this investigation four different strains of HSV-1 due to varying degrees of pathogenesis in mice [29]. These included two high-passaged laboratory strains, F and KOS63, and two low-passaged clinical isolates [29], H299 and H129, of brain (herpes simplex encephalitis) [29] and eye (acute retinal necrosis) [30] origin.

**Figure 6:**
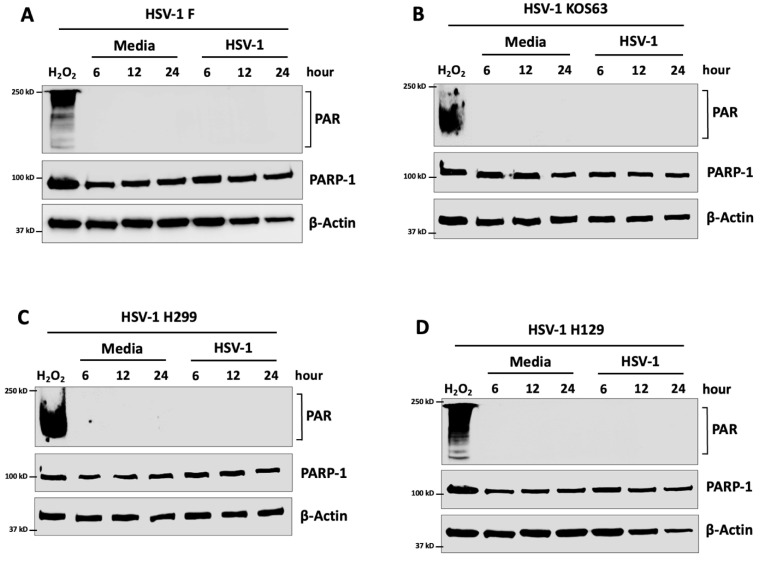
Monolayers of ARPE-19 cells were either mock-infected with cell culture maintenance medium (control) or infected with (**A**) HSV-1 F strain, (**B**) HSV-1 KOS63 strain, (**C**) HSV-1 H299 strain, or (**D**) HSV-1 H129 strain at moi = 5 PFU/cell. At 6, 12, and 24 h postinfection, all monolayers were collected and assessed by Western blot analysis for detection of PAR protein, PARP-1 protein, and β-Actin protein (internal control). Uninfected ARPE-19 cells were treated with H_2_O_2_ (500 uM) contained in DMEM for 15 min prior to harvesting to induce oxidative stress [27] and PAR protein as a positive control.

Monolayers of ARPE-19 cells were either mock-infected or infected with one of the four strains of HSV-1 (moi = 10), harvested at 6, 12, and 24 h postinfection, and subjected to Western blot analysis for detection of PAR protein and PARP-1 protein. Included in these experiments were uninfected ARPE-19 cells treated with H_2_O_2_ to induce PAR protein and PARP-1 protein production via oxidative stress to serve as a positive control. As shown in Figure 6, H_2_O_2_-treated uninfected ARPE-19 cells consistently displayed both PAR protein and PARP-1 protein production to show that this cell type possessed the cellular machinery to produce these parthanatos-associated proteins upon oxidative stress. In comparison, however, PAR protein was not detected in mock-infected nor HSV-1-infected ARPE-19 cells at all times examined (Figure 6). Moreover, findings were not virus strain dependent; the same outcome was observed when infected with any of the four different HSV-1 strains.

## 4. Discussion

Parthanatos as a newly recognized PCD signaling pathway is relatively unexplored. Nonetheless, its operation in many disease states has become more appreciated in recent years. This unique form of PCD has been identified in a wide variety of different diseases that have included several cancers (breast cancer, colon cancer, ovarian cancer, oral squamous cell carcinoma, and melanoma), diabetes, renal diseases, cardiovascular diseases, and neurodegenerative diseases [35,36,37,38]. In addition, parthanatos is gaining attention in ophthalmology in relationship to the pathogenesis of retinal diseases [39]. These include glaucoma, diabetic retinopathy, and age-related macular degeneration [40,41]. We have extended this list to possibly include AIDS-related HCMV retinal necrosis [23].

Parthanatos is triggered by excessive DNA damage that is often due to oxidative stress induced by high ROS levels [9,10,11,12,13]. Active virus replication also promotes ROS production [42] as shown for the replication of HCMV in human fibroblasts [43] and several human hematopoietic cell lines [33]. In fact, HCMV induces ROS generation within minutes after virus entry into human smooth muscle cells [44]. Despite these studies, there has been a surprising lack of information on parthanatos during cytomegalovirus replication at the cellular level. Toward this end, we compared three diverse cell types (mouse fibroblasts, mouse lung cells, and mouse microglial cells) for the detection of PAR protein as a marker for early parthanatos induction [13] and discovered some unexpected cell type differences. Firstly, MCMV-infected MEF cells were found to produce PAR protein in a virus dose-dependent manner. Secondly, stimulation of PAR protein was found to be cell type dependent. Unlike MCMV-infected MEF cells, MCMV-infected MLg cells failed to show detectable amounts of PAR protein at all times examined, even at the highest input dose of infectious virus. This outcome may be due to this cell type lacking the cellular machinery to undergo parthanatos, as suggested by the inability of H_2_O_2_ treatment as a positive control to stimulate PAR protein production. Thirdly, MCMV infection of BV-2 cells resulted in a pattern of PAR protein production different from that observed for MCMV-infected MEF cells or MLg cells. Whereas PAR protein was reproducibly detected at 3 days after infection with MCMV at a low input dose of infectious virus (moi = 1), PAR protein was not detected at higher input doses of infectious virus (moi = 5 and 10) at all times examined. In sharp contrast, PARP-1 protein was detected in all three host cell types, whether MCMV-infected or mock-infected. Indeed, the continuous detection of PARP-1 protein within all three host cell types served as a positive control for these studies due to its role as a “genome guardian” [13] involved in repair of minor DNA damage.

We also provide new evidence that induction of parthanatos-associated PAR protein in MCMV-infected MEF cells does not involve structural tegument proteins of the parental virion released into the host cell after attachment and adsorption. Many tegument proteins of cytomegaloviruses are structural phosphoproteins [45]. These play key roles in the activation of virus-encoded immediate-early gene expression during the initiation of productive virus replication [13]. We were therefore especially curious about this possibility due to the work of Kim et al. [33], who identified at least one phosphoprotein, pp65, involved in the induction of parthanatos in HCMV-infected HEL 299 cells. Instead, we found that PAR protein was not induced in MEF cells inoculated with UV-inactivated parental virus. This outcome suggests the production of immediate-early, early, and/or late gene expression during active virus replication is indeed required for PAR-associated parthanatos induction. This conclusion is in agreement with those of Kim et al. [33] who linked parthanatos induction to several HCMV gene products produced during the course of productive virus replication. These included IE1 and IE2 immediate-early proteins and UL33 early protein. MCMV homologues to these HCMV-induced proteins, however, would not be produced in MEF cells after inoculation with UV-inactivated parental virus particles and would be deficient in all kinetic classes of virus gene expression [46] due to DNA-damaging UV light [34]. It is noteworthy that PARP-1 protein production within MEF cells remains unaffected when inoculated with either infectious MCMV or UV-inactivated MCMV, suggesting that PARP-1 protein production within MEF cells is not influenced by virus inoculation and replication.

Although parthanatos has been recognized as a PCD signaling pathway, the few investigations exploring its participation during virus infection have only demonstrated virus-induced induction of the pathway and ultimately cell death, an outcome that would be detrimental to virus survival. We observed this apparent outcome in MEF cells infected with MCMV. Cytomegaloviruses, however, encode for proteins that suppress rather than induce other host cell death signaling pathways, a mechanism to ensure virus survival. Specifically, individual MCMV and HCMV-encoded proteins have been identified that suppress extrinsic apoptosis, necroptosis, and canonical pyroptosis signaling pathways and, in this manner, counteract host cell death [18]. That BV2 cells in the present investigation exhibited apparent suppression of parthanatos-associated PAR protein with increased amounts of infectious virus may be the first evidence that a virus, in this case MCMV, can suppress parthanatos-induced host cell death in some cell types. This could possibility occur through a mechanism involving one or more virus-encoded proteins as observed for other PCD pathways. Studies are in progress to explore this intriguing hypothesis.

Because a majority of our studies focused on MCMV induction of PAR protein as a marker for an early parthanatos pathway event, especially when comparing MEF cells with MLg cells that yielded markedly different outcomes, we sought to also compare these two cell types for a late parthanatos pathway event. For this study, we chose AIF. Although AIF is naturally found within the inner mitochondrial membranes where is serves to stabilize mitochondrial structure and plays an important role in cell survival [13], AIF has also been identified as an essential participant in the parthanatos pathway. Upon translocation of PAR protein from the nucleus to mitochondria of the cytoplasm, PAR polymers are thought to induce AIF release from mitochondria and its subsequent redistribution into the nucleus where chromatin condensation, DNA fragmentation, and cell death ensue. This series of events already established by others for the parthanatos pathway [7,8,9,10,11,12,13] prompted subcellular fractionation studies by us to trace the movement of AIF from mitochondria to nucleus in either MEF cells or MLg cells infected with MCMV. As expected, AIF was detected in the mitochondrial fraction of both cell types following either MCMV infection or mock infection due to its service as a structural component of mitochondria. Surprisingly, AIF was detected within nuclear fractions of both MEF cells and MLg cells infected with MCMV at 3 days after inoculation, even though PAR protein was not detected in MCMV-infected MLg cells at all times examined. Although detection of AIF within nuclear subcellular fractions of MCMV-infected cells might imply subsequent cell death due to DNA fragmentation, it should be noted that AIF does not exhibit endonuclease activity [8] and suggests the need for the participation of another protein with nuclease activity for PCD. This additional protein may be migration inhibitory factor (MIF). Initially identified as a trigger and amplifier for production of several proinflammatory cytokines associated with innate immunity [47], subsequent investigations demonstrated that MIF also possesses nuclease activity [48] and may therefore function during parthanatos as a downstream nuclease effector of cell death [12]. In fact, evidence has emerged to suggest that AIF couples with MIF in the cytoplasm and serves as a chaperone molecule to translocate MIF to the nucleus as a complex to initiate AIF-associated DNA nuclease activity [13] (Figure 1). Thus, detection of AIF alone within the nucleus of MCMV-infected cells does not necessarily imply the operation of parthanatos that may be dependent on the concomitant participation of MIF.

Given the similarities between apoptosis and parthanatos whose ultimate cell death mechanisms are DNA fragmentation but to differing degrees, MIF also inhibits apoptosis via initiation of the EFK1/2 MAP kinase pathway [13]. This observation suggests that caspase-dependent apoptosis and caspase-independent parthanatos as distinct forms of PCD pathways cannot operate simultaneously within the same MCMV-infected host cell and collaborate toward cell death. It is also possible that noncanonical forms of parthanatos may be operating in MCMV-infected cells and help explain some of our surprising findings. For example, an AIF-independent PARP-1-dependent cell death has been reported to be a major mechanism by which retinal degeneration occurs during the pathogenesis of dry age-related macular degeneration [41].

We also included HSV-1 in our investigations of parthanatos. Like cytomegaloviruses, HSV-1 replication has been shown to stimulate ROS in several cell types including neural cell types [49,50,51]. We therefore explored possible HSV-1-induced stimulation of parthanatos-associated PAR protein in ARPE-19 cells derived from RPE cells of the retina [22], because HSV-1 has been shown to be an etiologic agent for acute retinal necrosis in healthy persons [30]. Unlike MCMV infection of MEF cells where PAR protein stimulation was demonstrated, PAR protein stimulation was not detected in HSV-1-infected ARPE-19 up to 48 h after virus infection. This finding was confirmed after infection with any one of four different strains of HSV-1 that included two clinical isolates, one recovered from the eye of an adult patient with ARN (H299) and one recovered from the brain of an adult patient with fatal herpes simplex encephalitis (H129). These findings, however, are at odds with those reported previously by Grady et al. [27], who showed that HSV-1 infection of primary human fibroblasts resulted in the stimulation of PARP-1 protein and PAR protein in response to DNA damage. However, their study and our study used the F strain of HSV-1, ruling out a strain-dependent difference in results. It is therefore possible that HSV-1 like MCMV exhibits a cell type-dependent difference in the stimulation or suppression of the parthanatos PCD signaling pathway.

The results reported herein greatly broaden our understanding of parthanatos as a recognized form of PCD with special attention to MCMV and HSV-1 replication. Collectively, our findings suggest that the operation of parthanatos as a relatively new addition to the growing PCD family is far more complex at the virus–host cell level than previously thought. Its stimulation in response to MCMV infection is surprisingly cell type specific, an observation than may also extend to HSV-1 infection. Perhaps more importantly is new data to support the idea that high dose MCMV infection may limit parthanatos-related signaling through virus suppression of the host cell death pathway. This hypothesis would align with work by others showing that cytomegaloviruses [18], as well as HSV-1 [19], encode for multiple suppressors of PCD, including inhibitors of apoptosis, necroptosis, and pyroptosis, which help to preserve the infected cell for efficient productive virus replication. What remains unclear, however, are the virus and/or host cell factors that determine whether parthanatos is induced to clear virus replication or suppressed to allow virus replication and therefore act as a traditional PCD during virus infection. What is also unclear is the relative role of parthanatos in the onset and development of retinal diseases of herpesvirus origin. Toward this end, we have been using mice with MAIDS-related MCMV retinal necrosis [24,25,26] as an experimental model of AIDS-related HCMV retinal necrosis to explore the relative participation of parthanatos, as well as other forms of PCD [52,53,54,55], during retinal disease pathogenesis.

Previous work by us reported that whole MCMV-infected eyes of mice with MAIDS exhibit significant stimulation of detectable PARP-1 protein and PAR protein during the onset and development of retinal necrosis [23]. Nonetheless, we also reported the significant intraocular stimulation of poly(ADP-ribose) glycohydrolase (PARG), which functions as a PAR-degrading enzyme [56,57]. PARG protein has been shown to inhibit parthanatos through PAR polymer degradation by dampening its toxic accumulation, prevent mitochondrial release and translocation of AIF (and possibly MIF) to the nucleus, and ultimately prevent cell death. That parthanatos can be naturally inhibited by PARG suggests potential targets for therapeutic intervention to manage herpesvirus-induced retinal diseases, such as AIDS-related HCMV retinal necrosis and possibly HSV-1-induced acute retinal necrosis. For example, Iduna protects against glutamate excitotoxicity and stroke in mice by binding to the PAR polymer and, in this manner, abolishes parthanatos-induced cell death [58]. PARP-1 overactivation is another potential therapeutic target. Indeed, several PARP-1 inhibitors have been developed to inhibit DNA damage repair and promote cell death for several forms of cancer [59,60]. These include olaparib, ruparib, niraparib, and talazoparib [61,62,63,64,65]. Whether therapeutic intervention directed at PAR protein and/or PARP-1 protein might affect the natural course of MAIDS-related MCMV retinal necrosis is presently being investigated.

## 5. Conclusions

Parthanatos is a novel PARP-1-dependent, caspase-independent PCD cascade mediated by mitochondrial AIF with possible MIF involvement, resulting in large-scale DNA fragmentation and cell death in response to oxidative stress, which can be induced by virus infection. Investigation of parthanatos vis-à-vis MCMV or HSV-1 infection of several diverse cell types has revealed that induction of parthanatos is surprisingly cell type specific. We also provide new evidence to suggest that productive replication of MCMV and HSV-1 may alternatively suppress parthanatos in some cell types, and that, in the case of HSV-1, suppression of parthanatos is not virus strain dependent. We conclude that the operation of parthanatos as a PCD signaling pathway at the host cell level during herpesvirus replication is more complex than originally thought. Nonetheless, further investigation of parthanatos during virus infection may offer new targets for possible therapeutic interventions.

## Figures and Tables

**Table 1:** Comparison of apoptosis, pyroptosis, and necroptosis with parthanatos as cell death signaling pathways.

	Signaling Pathway	Proinflammatory?
**Apoptosis ^a^**	TNF	No
	TNFR1	
	Caspase-8	
	Caspase-3	
**Pyroptosis ^b^**	Inflammasomes	Yes
	Caspase-1	
	Gasdermin D	
**Necroptosis**	RIPK1	Yes
	RIPK3	
	MLKL	
**Parthanatos**	PARP-1	No
	PAR	
	AIF	

^a^ Extrinsic apoptosis. ^b^ Canonical pyroptosis.

## Data Availability

The original contributions presented in the study are included in the article. Further inquiries can be directed to the corresponding author.
